# *HIF-1α* Polymorphism in the Susceptibility of Cervical Spondylotic Myelopathy and Its Outcome after Anterior Cervical Corpectomy and Fusion Treatment

**DOI:** 10.1371/journal.pone.0110862

**Published:** 2014-11-17

**Authors:** Zhan-Chao Wang, Xu-Wei Hou, Jiang Shao, Yong-Jing Ji, Lulu Li, Qiang Zhou, Si-Ming Yu, Yu-Lun Mao, Hao-Jie Zhang, Ping-Chao Zhang, Hua Lu

**Affiliations:** 1 Department of Orthopedics, Xinhua Hospital (Chongming), Shanghai Jiaotong University, Shanghai, China; 2 Department of Cardiology, Hangzhou Hospital, Nanjing Medical University & Hangzhou First Municipal Hospital, Hangzhou, China; 3 Department of Orthopedics, Xinhua Hospital, Shanghai Jiaotong University; Shanghai, China; 4 Department of Internal Medicine, Jinan 2nd People's Hospital, Jinan, China; 5 Department of Biostatistics, College of Public Health, Harbin Medical University, Harbin, Heilongjiang, 150081, China; Ohio State University Medical Center, United States of America

## Abstract

**Background:**

To investigate the association between the single nucleotide polymorphism (SNP) of hypoxia-inducible factor1 α *(HIF-1α)* and the susceptibility to cervical spondylotic myelopathy (CSM) and its outcome after surgical treatment.

**Method:**

A total of 230 CSM patients and 284 healthy controls were recruited. All patients received anterior cervical corpectomy and fusion (ACF) and were followed for 12 months. The genotypes for two *HIF-1α* variants (*1772C>T and 1790G>A*) were determined.

**Results:**

In the present study, we found that the *HIF-1α* polymorphism at *1790G>A* significantly affects the susceptibility to CSM and its clinical features, including severity and onset age. In addition, the *1790A>G* polymorphism also determines the prognosis of CSM patients after ACF treatment. The *GG* genotype of *1790G>A* polymorphism is associated with a higher risk to develop CSM, higher severity and earlier onset age. More importantly, we found that the *1790G>A* polymorphism determines the clinical outcome in CSM patients who underwent ACF treatment.

**Conclusion:**

Our findings suggest that the *HIF-1α 1790G>A* polymorphism is associated with the susceptibility to CSM and can be used as predictor for the clinical outcome in CSM patients receiving ACF treatment.

## Introduction

Degenerative changes in the cervical spine are an inevitable response to the aging process. Impairment of cervical nerve roots may result from instability, disc degeneration, herniation or spinal stenosis. Cervical spondylotic myelopathy (CSM) is one of the most common degenerative spinal cord disorders affecting the elderly [1], [2], [3]. The mechanism of CSM development remains unclear. Some environmental factors, such as age, gender, smoking and trauma are reported to be associated with CSM risk [4], [5]. Previous studies show that the genetic factors also play an important role in the CSM development [6], [7]. Some candidate genes predicting the occurrence and development of CSM have been reported [8], [9]. Anterior cervical corpectomy and fusion (ACF) is a widely used surgical treatment for CSM patients. A recent study shows that the patient's genetic background affects the clinical outcome of CSM patients receiving ACF treatment [10].

The effect of hypoxia on the development of chronic spine disease has aroused interest. Hypoxia differentially regulates human nucleus pulposus and annulus fibrosus cell extracellular matrix production in 3D scaffolds [11]. As the largest avascular structure in the body, intervertebral disc is characterized by low oxygen tension *in vivo* [12]. Hypoxia-inducible factor α (HIF-1α) is a master transcription factor that regulates the cellular responses to hypoxic environments. HIF-1α is expressed in nucleus pulposus cells and plays an important role in regulating energy metabolism and matrix synthesis [13], [14], [15]. A recent study revealed that HIF-1α plays a crucial role in the survival of disc cells and resorption of the herniated disc in human [16]. HIF-1α is involved in the homeostasis of intervertebral disc cells. HIF-1α regulates apoptosis of intervertebral disc cells [16] [26].

Two *HIF1α* polymorphisms, namely, *1772C>T (P582S)* and *1790G>A (A588T)* have been reported to significantly increase *HIF1α* gene transcriptional activity [17], [18]. A recent study suggests that *HIF-1α* polymorphism affects lumbar disc degeneration and confers the susceptibility to lumbar disc disease (LDD) in Chinese cohort [19]. To date, the role of *HIF-1α polymorphism* in CSM remains unknown. In this study, we enrolled the Chinese CSM patients to investigate the association of *HIF-1α* polymorphism with the susceptibility, clinical feature and prognosis of CSM patients after ACF treatment.

## Methods

### Ethics statement

The ethical committee of Shanghai Jiaotong University approved the study. All participants provided their written informed consent to participate in this study.

### Enrolment

In our study, the sample size required to achieve statistically significant associations were calculated using the power calculator for case control genetic association studies (PGA). According to the estimated sample size, we enrolled 230 patients with CSM. The diagnose was established on the basis of findings from the history, physical examination and confirmed by magnetic resonance imaging (MRI). Patients with one of the following conditions were excluded from this study: cervical trauma, autoimmune disease, chronic inflammatory disease, severe osteoporosis, and chronic renal or liver insufficiency. The control group consisted of 288 sex and age matched healthy Chinese individuals. All controls underwent the MRI and show no evidence of spondylosis, cord or nerve root compression and osteophyte formation in spine. The clinical characteristics including sex, age, weight, height, body mass index (BMI), daily desk work time, smoking status and family history of intervertebral degenerative disc disease were collected. The severity of CSM was scored according to the modified Japanese Orthopedic Association (modified JOA) score for CSM [20].

### Follow-up

All 230 patients received anterior cervical corpectomy and fusion (ACF) and were followed for 2 years. The patients were dichotomized into two groups according to the mJOA scores: improvement group (at least 50% or higher improvement in mJOA score at the last follow-up compared with pre-operative score) and a non-improvement group (the improvement of mJOA score at last follow-up was less than 50%, equal, or less than pre-operative mJOA score) [10].

### *HIF-1α* genotyping

Genomic DNA was isolated from the peripheral blood leukocytes by using standard protocols. Polymerase chain reaction (PCR) was performed to amplify the 178-bp fragment of the exon 12 of the *HIF-1α* human gene, using the 5′-CAT GTA TTT GCT GTT TTA AAG-3′ forward primer and 5′-GAG TCT GCT GGA ATA CTG TAA CTG-3′ reverse primer. The mixture for PCR was in 30 µL, containing 200 ng template DNA, 0.2 mM of each dNTP, 0.5 µM of each forward and reverse primer, 1.5 mM MgCl2, 0.5 U of Taq polymerase and 3 µL of 10× PCR buffer. The conditions for the PCR reaction were: denaturation at 95°C for 5 min, followed by 35 cycles of denaturation at 95°C for 30 sec, annealing at 61°C for 30 sec, extension at 70°C for 1 min, and a final extension at 72°C for 10 min. PCR products were purified and sequenced using Big Dye Terminator kit on an ABI Prism 3100 Automated DNA sequencer according to the manufacturer's protocol (Applied Biosystems, Foster City, CA).

### Western blot assay

The intervertebral discs were collected during surgery from patients during ACF treatment. Samples were homogenized and lysed. Extracts were resolved on SDS-polyacrylamide gels followed by transfer to nitrocellulose membranes. Proteins were resolved by electrophoresis on 8–12% sodium dodecyl sulfate–polyacrylamide gels and transferred by electroblotting to polyvinylidene difluoride membranes. The membranes were blocked with 5% nonfat dry milk and incubated overnight at 4°C with the anti-HIF-1α (Novus Biological, 1;1000), anti-vascular endothelial growth factor (anti-VEGF) (Santa Cruz, 1∶1000), anti-VEGF receptor (anti-VEGFR) (Santa Cruz, 1∶1000), anti-NF-kB (Santa Cruz, 1∶1000), anti-interleukin 1 (anti-IL1) (Santa Cruz, 1∶1000), anti-interleukin6 (anti-IL6) (Santa Cruz, 1∶1000), anti-Osteopontin (OPN) (Santa Cruz, 1∶1000), anti-Osteoprotegerin (OPG) and anti-GAPDH (Santa Cruz, 1∶2000), antibodies. Immunolabeling was detected using the enhanced chemiluminescence Reagent (Amersham Biosciences).

### Statistical analysis

Data on quantitative characteristics are expressed as means ± SD. Data on qualitative characteristics are expressed as percent values or absolute numbers, as indicated. Differences in demographic characteristics and vascular risk factors between patients and controls were compared by using Student's t test or ANOVA for continuous variables and the χ2 test for all categorical variables. To estimate the deviation of frequency of gene alleles in tested population, we performed the Hardy-Weinberg equilibrium using χ2 tests. Genotypes and allele frequencies were compared by χ2 analysis or Fisher's exact test. Multivariate logistic regression analysis was used to determine the influence of *HIF-1α* polymorphism on CSM, controlling potential confounding conventional risk factors. A forward stepwise (Likelihood Ratio) procedure was used for multivariable analysis. Data were analyzed with the SPSS 16.0 package (Statistical Package for the Social Sciences, version 16.0, SPSS Inc, Chicago, IL, USA). The results were considered statistically significant at P<0.05 using a 2-tailed test.

## Results

Table 1 shows the clinical characteristics of CSM patients and controls. There was no significant difference in age, sex and BMI between two groups. However, CSM patients had a significantly higher rate of smoker, family history for spine disorders, Diabetes mellitus (DM) and daily desk work time than controls (all P<0.001).

**Table 1:** Characteristics of subjects.

*Variables*	*CSM patients*	*Controls*	*P value*
*Age(mean ± SD)*	*45.3±4.4*	*45.2±2.5*	*0.853*
*Gender (Male,%)*	*57.4*	*58.1*	*0.654*
*BMI(mean ± SD)*	*23.2±2.3*	*23.1±2.5*	*0.753*
*Smoker (%)*	*35.3*	*20.5*	*<0.001*
*DM*	*21.3*	*9.5*	*<0.001*
*Spine disorder family history (%)*	*20.5*	*7.8*	*<0.001*
*Desk worktime (hour/d)*	*5.5±1.2*	*3.6±0.9*	*<0.001*
*Operation cervical segment number*			
*1*	*156*		
*2*	*54*		
*3*	*20*		

Table 2 describes the genotype distributions and allele frequencies of *HIF-1α* polymorphisms in CSM and control subjects. The genotype frequencies for both polymorphisms were not significantly different from those expected under Hardy–Weinberg equilibrium (all P>0.05). There were no significant difference in the *1772C>T* genotypes between CSM patients and controls. For the *1790G>A* polymorphism, the CSM patients had a significant higher prevalence of *GG* genotype than controls (29.13% % vs. 17.96%, P<0.001). To determine the independent risk factor for CSM, we preformed the multivariate logistic regression analysis with the adjustment of age, sex, BMI, smoking status, family history status and daily desk work time. With the *1790AA* genotype as reference, our data showed that the *1790GG* genotype carriers had a higher risk for CSM development (adjust OR = 2.37, 95%CI: 1.47–3.83, adjusted P<0.001). The *1790G* allele also represented a higher risk for CSM (adjusted OR = 1.62, adjusted P<0.001). In contrast, the *1772 C>T* polymorphism did not affect the risk for CSM in our study.

**Table 2:** The genotype and allele frequencies of HIF-1α polymorphism in CSM and control subjects.

*Genotype*	*CSM (n)*	*%*	*Control(n)*	*%*	*adjusted OR*	*95%CI*		*adjusted P*
*1790AA*	*62*	*26.96%*	*112*	39.44%	*1.00*			
*1790GA*	*101*	*43.91%*	*121*	42.61%	*1.51*	*1.00*	*2.27*	*0.07*
*1790GG*	*67*	*29.13%*	*51*	17.96%	*2.37*	*1.47*	*3.83*	*<0.001*
*A*	*225*	*48.91%*	*345*	60.74%	*1.00*			
*G*	*235*	*51.09%*	*223*	39.26%	*1.62*	*1.26*	*2.07*	*<0.001*
*1772CC*	*89*	*20.41%*	*84*	15.79%	*1.00*			
*1772CT*	*104*	*23.85%*	*146*	27.44%	*0.67*	*0.46*	*0.99*	*0.18*
*1772TT*	*37*	*8.49%*	*54*	8.27%	*0.79*	*0.47*	*1.35*	*0.21*
*C*	*442*	*50.69%*	*526*	49.44%	*1.00*			
*T*	*430*	*49.31%*	*538*	50.56%	*0.95*	*0.80*	*1.14*	*0.76*

**Figure 1:**
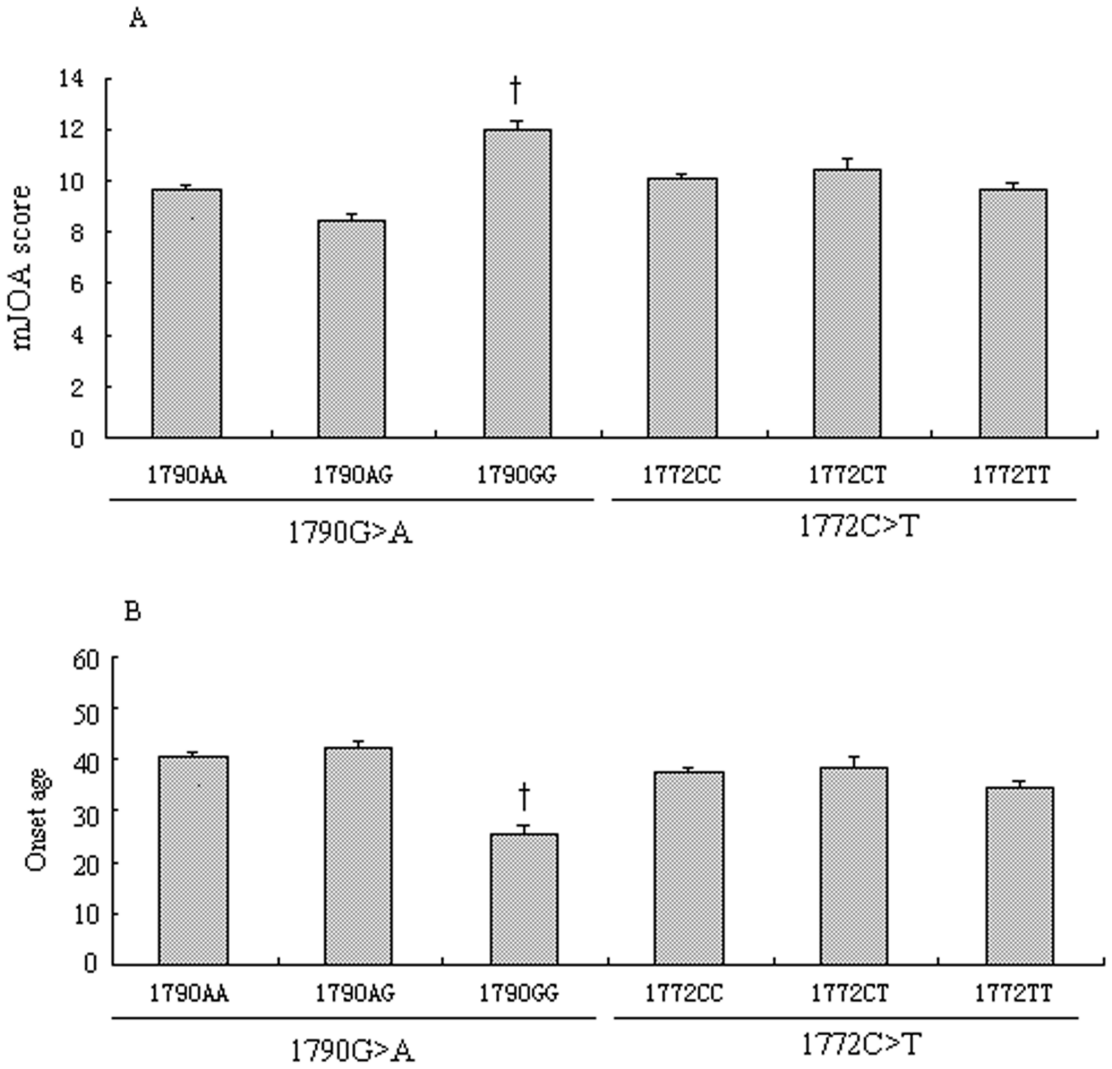
HIF-1α polymorphisms with the clinical features of CSM patients. Figure 1 shows that the 1790G>A dramatically affects the severity (Figure 1A) and onset age (Figure 1B) of CSM patients. Patients with the 1790GG had a higher mJOA score (Figure 1A) and earlier on set age (Figure 1B) than those with 1790GA and 1790AA genotypes (†, P<0.001).

Among all CSM patients, we evaluated the association between the *HIF-1α* polymorphisms and the clinical features of CSM patients before their surgical treatment. The *1790G>A* and *1772C>T* did not affect the smoking status, daily desk work time and family history status. However, we found the *1790G>A* polymorphism dramatically affects the severity and onset age of CSM patients. The *1790GG* patients had higher mJOA score (Figure 1A) and earlier on set age (Figure 1B) than *1790GA* and *1790AA* carriers (†, P<0.001).

**Figure 2:**
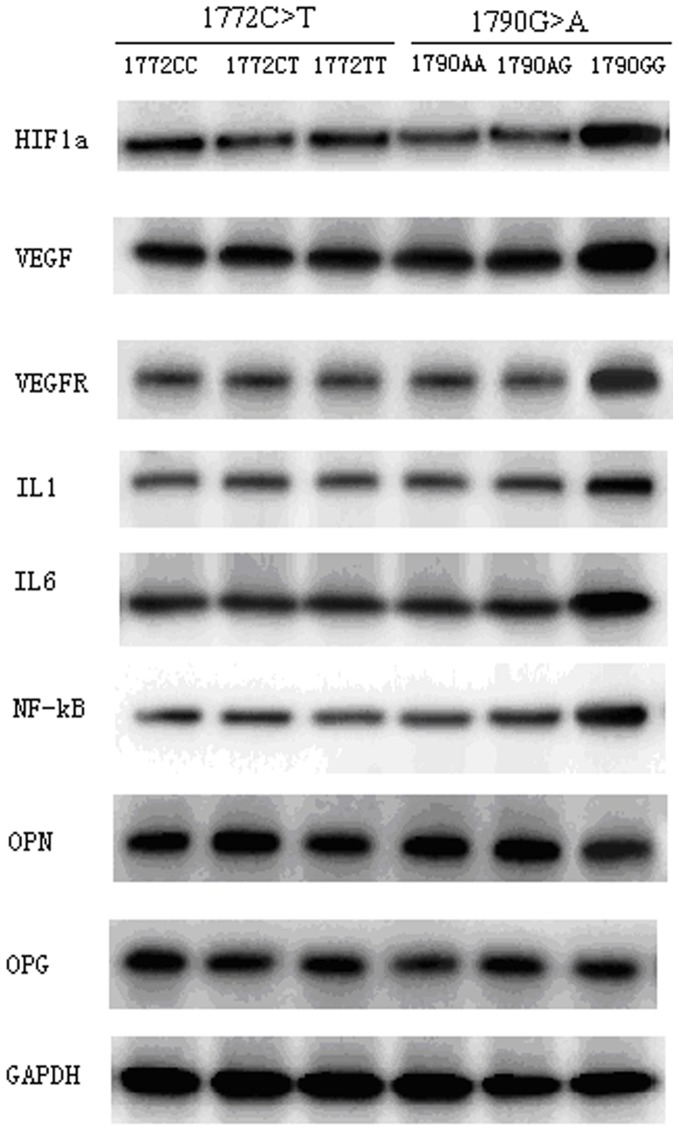
The protein expressions of HIF-1α, VEGF, VEGFR and a series of inflammatory factors based on HIF-1α polymorphisms. Figure 2 shows that only the 1790A>G polymorphism significantly affects the expression level of HIF-1α, VEGF, VEGFR, IL1, IL6 and NF-kB protein expressions compared to 1970AA and 1970AG. The OPG and OPN levels were not changed when stratified by 1790A>G polymorphism (Figure 2). The 1772C>T genotype did not influence the above mentioned factors expression levels.

We next compared the protein expressions of HIF-1α, VEGF, VEGFR and a series of inflammatory factors in disc samples from CSM patients (Figure 2). We found that only the *1790A>G* polymorphism significantly affected the above mentioned factor expression levels (Figure 2). The *1790GG* genotype carriers had higher levels of HIF-1α, VEGF, VEGFR, IL1, IL6 and NF-kB compared to the *1970AA* and *1970AG* carriers, but did not affect the OPG and OPN levels (Figure 2). In contrast, the *1772C>T* genotype did not influence any of the above mentioned factors expression levels.

All CSM subjects receiving ACF treatment were alive and completed the 12 months follow-up. All patients According to the modified JOA scores, 147 patients were attributed into improvement group and 83 into non-improvement groups. Again, we found that the *1790A>G* polymorphism distribution were significantly different between the improvement and non-improvement groups. The *1790GG* genotype was more prevalent in CSM patients with poor outcome than those with good outcome (Table 3). Multiple logistic regression analysis showed the *1790GG* polymorphism was associated with higher risk for a poor outcome (non-improvement) after ACF treatment (adjusted OR = 2.66, adjusted P = 0.019, compared to *1790AA* genotype).

**Table 3:** The effect of genotype distributions and allele frequencies of HIF-1α polymorphisms on the clinical outcome after ACF treatment.

*Genotype*	*Non-Improvement*		*Improvement*		*Adjusted OR*	*95%CI*		*Adjusted P*
*1790AA*	*15*	*18.07%*	*34*	*23.13%*	*1.00*			
*1790AG*	*41*	*49.40%*	*90*	*61.22%*	*1.03*	*0.51*	*2.10*	*0.930*
*1790GG*	*27*	*32.53%*	*23*	*15.65%*	*2.66*	*1.17*	*6.06*	*0.019*
*A*	*71*	*42.77%*	*158*	*53.74%*	*1.00*			
*G*	*95*	*57.23%*	*136*	*46.26%*	*1.55*	*1.06*	*2.28*	*0.024*
*HIF-1α*								
*Low*	*25*	*30.12%*	*77*	*52.38%*	*1.00*			
*High*	*58*	*69.88%*	*70*	*47.62%*	*2.55*	*1.44*	*4.51*	*0.001*
*VEGF*								
*Low*	*28*	*33.73%*	*81*	*55.10%*	*1.00*			
*High*	*55*	*66.27%*	*66*	*44.90%*	*2.41*	*1.38*	*4.22*	*0.002*
*VEGFR*								
*Low*	*37*	*44.58%*	*95*	*64.63%*	*1.00*			
*High*	*46*	*55.42%*	*52*	*35.37%*	*2.27*	*1.31*	*3.93*	*0.003*

## Discussion

In the present study, we found that the *HIF-1α* polymorphism at *1790G>A* significantly affects the susceptibility to CSM and is associated with its clinical features in CSM patients, including the severity and the onset age. In addition, the *1790A>G* polymorphism also determines the prognosis of CSM patients after ACF treatment. The *GG* genotype of *1790G>A* polymorphism is associated with higher risk to develop CSM, higher severity and earlier onset age. This genotype also presents a higher possibility for a poorer clinical outcome after CAF treatment. Our findings suggest that the *HIF-1α* polymorphism at *1790G>A* may be used as a molecular marker for the CSM.

Hypoxia is a main characteristic of bone diseases like osteonecrosis and osteoarthritis [21], [22] [23]. HIF-1α is the major transcriptional regulator triggered in hypoxia to promote adaptation to the new environment. Under normal oxygen conditions, HIF-1α is continuously produced and destroyed. However, under hypoxic conditions, the expression of HIF-1α is stabilized and translocates to the nucleus where it dimerizes with HIF-1β, thus promots the transcription of its target genes, including VEGF [24], [25], [26] [10].

Several studies have shown that HIF1α plays an important role in growth plate morphogenesis, fracture healing, and distraction osteogenesis [25], [27], [28], [29]. To date, little is know about the association of *HIF-1α* polymorphism and bone disorders. In a previous study, the *HIF1α* polymorphism at *+45319C>T* (the *1772C>T* in our study) and several other loci are associated with idiopathic osteonecrosis of the femoral head (ONFH) in Korean men [22], [30], [31], suggesting that *HIF1α* variations play a role in the pathogenesis of ONFH. However, in our current study, the *HIF1α* polymorphism at *+45319C>T* was not associated with the CSM susceptibility in Chinese patients. In contrast, another SNP at locus, *1790A>G* was shown to be closely related to the risk, severity, onset age of CSM patients. Also it should be noted that the *HIF1α* polymorphisms distribution was quite different from Koreans and Chinese based on the genotype distribution data from their study and ours. Our results are consistent with another study in Chinese patients, in which the authors found that the *1790A>G* polymorphism affects the risk and severity of lumbar disc degeneration (LDD) [19].

To date, only one study reported that the association of the gene polymorphism of a candidate gene with the clinical outcome of surgical treatment of ACF [10]. Bone morphogenic proteins-4 (BMP-4) polymorphism is associated with the functional improvement from ACF surgery [10]. In our study, we found that the *1790A>G* polymorphism determines clinical improvement of CSM patients after ACF treatment. Our findings suggest that the *HIF-1α* polymorphism at *1790G>A* be used as a prognostic marker for the CSM underwent ACF treatment.

In hypoxic condition, the up-regulation of VEGF is consistent with increasing HIF-1α in acute periods. HIF-1α/VEGF signaling pathway is thought to play a dual role following acute spinal cord injury [32], [33]. In the present study, we found that the HIF-1α 1790G>A influences local expression of VEGF and VEGFR in cervical disc tissues. The 1790GG genotype carriers tend to have higher HIF-1α and VEGF expressions, which is consistent with a previous study [19]. In addition, we observed higher expressions of VEGFR, NF-KB, IL1 and IL6. However, the OPN and OPG levels were not affected by 1790A>G polymorphism. We postulate that the 1790A>G polymorphism may affect the local inflammation level in the intervertebral discs among patients with different genotype carriers, thus confers the susceptibility to CSM in these patients.

Some limitations in this study should be addressed. First, this was a single-center based study and only Chinese patients were enrolled. Thus the findings of this study need validation by another duplicate study. Secondly, we did not illustrate the mechanism under which the *HIF-1α* gene polymorphism affects CSM development.
